# Clinical characteristics, genomic profiling and outcomes of single system multifocal Langerhans cell histiocytosis in adults with bone involvement

**DOI:** 10.1038/s41408-023-00913-8

**Published:** 2023-09-05

**Authors:** Hong-xiao Han, Long Chang, Min Lang, He Lin, Jian Li, Ming-hui Duan, Dao-bin Zhou, Xin-xin Cao

**Affiliations:** 1 Department of Hematology, Peking Union Medical College Hospital, Chinese Academy of Medical Sciences & Peking Union Medical College, Beijing, China; 2 State Key Laboratory of Complex Severe and Rare Diseases, Peking Union Medical College Hospital, Chinese Academy of Medical Sciences & Peking Union Medical College, Beijing, China

**Keywords:** Haematological cancer, Cancer genetics

Dear Editor,

Langerhans cell histiocytosis (LCH) is a rare, heterogeneous histiocytic disease derived from the misguided clonal expansion of CD1a-positive and CD207-positive myeloid precursors cells [1]. LCH can affect individuals of all ages; however, it is more commonly in children [2]. The clinical presentation can manifest as a benign unifocal single-system (SS-s) disease, a single system multifocal (SS-m) disease, or a multisystem (MS) disease with life-threatening organ failure [3]. SS-m disease is defined as having more than one lesion involved in any single organ [3]. The bone is the most commonly involved organ presented in approximately 80% of LCH patients [4]. Recurrent *BRAF*^*V600E*^ mutations were first identified in 57% of LCH samples in 2010 [5]. Subsequently, other *MAPK* pathway mutations have also been discovered [6, 7]. It has been reported that *BRAF*^*V600E*^ mutation occurred more frequently in pediatric patients than in adults [5]. However, no previous study has reported on the genetic mutations related to adult LCH with SS-m. Previous studies found that pediatric patients with central nerve system (CNS)-risk lesions involvement, which were defined as involving the craniofacial bone, orbital, ear, and oral structures, had a poor prognosis [8, 9]. Otherwise, the association between the outcome of LCH in adults with SS-m and CNS-risk lesions involvement has never been described.

To clarify these questions, we retrospectively studied 43 adult LCH (≥18 years) with SS-m at Peking Union Medical College Hospital between January 2001 and May 2023. The diagnosis of LCH was based on histological findings in accordance with the World Health Organization classification of hematopoietic neoplasms [10]. Patients with available tissues from the biopsy lesions underwent next-generation sequencing (NGS) of 183 genes to detected the presence of *MAPK* pathway mutations according to a previous described protocol (Table S1) [11]. The initial therapies included local therapy (radiation or surgery) or systemic therapies: cytarabine-based therapies [12]; vindesine and prednisone-based (VP-based) regimens [13]; and *BRAF* inhibitors. Overall survival (OS) was calculated from the diagnosis to the date of death or last follow-up. Progression-free survival (PFS) was defined as the diagnosis to the occurrence of disease progression, relapse, death from any cause or last follow-up. The last follow-up date was May 20, 2023. The study was performed in accordance with Helsinki’s declaration.

Out of 422 newly diagnosed LCH in adults, forty-three patients (10.2%) had SS-m LCH, and all of whom exhibited bone involvement. The baseline characteristics of the patients are summarized in Table S2. Twenty-seven patients were male, with an approximately male-to-female ratio of 1.7:1. The median age at diagnosis was 34 years (range, 21–65 years) and the median number of bone lesions was three (range, 2–11). Bone pain (90.7%) was the most common symptom, followed by tumor formation in a localized area (11.6%), toothache (4.6%), and hearing impairment (2.3%). The ribs (51.2%) were the most common site, followed by the pelvis (46.5%), spine (39.5%), skull (30.2%), maxillofacial bones (30.2%), limbs (27.9%), alveolar bone (4.6%), and sternum (2.3%) (Fig. S1). In addition, 23 patients (53.5%) had CNS-risk lesions involvement. Compared with the patients with SS-s of bone involvement (*n* = 53) in our whole cohort, those with SS-m had a significantly higher frequency of the pelvis (46.5% vs. 3.8%, *P* < 0.0001), spine (39.5% vs. 9.4%, *P* < 0.0001), skull (30.2% vs. 13.2%, *P* = 0.041), maxillofacial bone (30.2% vs. 3.8%, *P* = 0.001), and CNS-risk lesions involvement (53.5% vs. 22.6%, *P* = 0.002) (Table S3).

Twenty-eight patients (65.1%) had sufficient DNA from lesion tissues for NGS analysis. Seven patients (25.0%) had no pathogenic mutations. The median number of gene mutations was two (range, 1–9). Out of the 28 patients who underwent NGS, the overall percentage of *MAPK* pathway mutations was 64.3% (*n* = 18). The *BRAF*^*V600E*^ mutation and *BRAF*^*indel*^ (*BRAF*^*N486_T491 delinsK*^) were detected in 10 patients (35.7%), and one patient (3.6%), respectively. Other *MAPK* pathway mutations included *MAP2K1* (7.2%), *KRAS* (7.2%), *NRAS* (7.2%), *ERBB3* (7.2%), *TP53* (3.6%), *ARAF* (3.6%), *NF1* (3.6%), *VEGFA* (3.6%), and *KDR* (3.6%). Furthermore, the frequencies of *PIK3CA* and *PIK3R2* mutations were 3.6% (*n* = 1), and 3.6% (*n* = 1), respectively (Fig. 1).

**Fig. 1:**
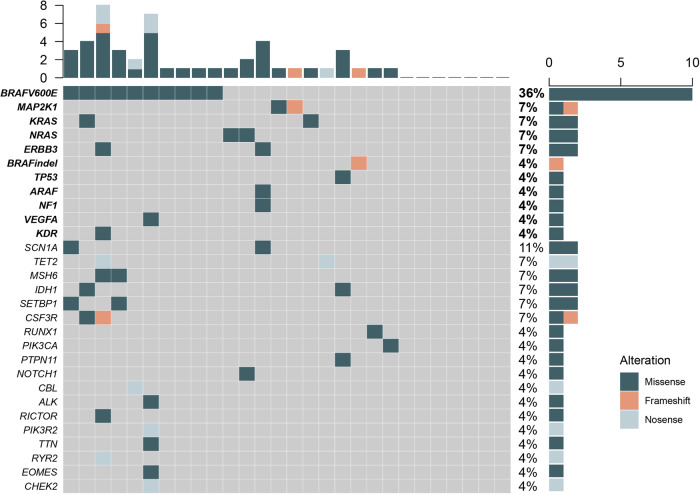
Mutational profiles of LCH in adults with single-system multifocal disease. Next-generation sequencing of lesion tissues of LCH in adults with single-system multifocal disease (SS-m).

The initial treatments are shown in Fig. 2A. Seven patients (16.3%) did not receive treatments. Local therapy was initiated in six patients (14.0%), including complete surgical resection (mandible and maxilla) in one patient, and surgery and postoperative radiation in five patients. Of the five patients who received postoperative radiation, four received postoperative radiation for all bone lesions, while one (mandible and ribs involvement) only received postoperative radiation for the mandible lesions. A total of 30 patients received first-line systemic treatment: 18 received cytarabine monotherapy, six received MA, four received VP-based regimens, and two who had CNS-risk lesions received *BRAF* inhibitors. After a median duration of 43 months follow-up (range, 1–218 months), one was lost to follow-up and one died from disease progression. The 3-year OS and PFS rates were 97.4% and 75.4%, respectively (Fig. 2B). Thirteen patients showed disease reactivation. All patients who received local treatment experienced disease reactivation, including three at original bone lesions, one at both original (right mandible and left pelvis) and new (right pelvis) bone lesions, and two who progressed to MS LCH. Both patients treated with *BRAF* inhibitors remained stable until the last follow-up. Univariate analysis showed that first-line systemic therapy compared to local therapy (36.3 months vs. 28.0 months, *P* = 0.013) and non-CNS-risk lesions involvement compared to CNS-risk lesions involvement (36.0 months vs. 23.0 months, *P* = 0.016) indicated better PFS (Fig. 2C, D).

**Fig. 2:**
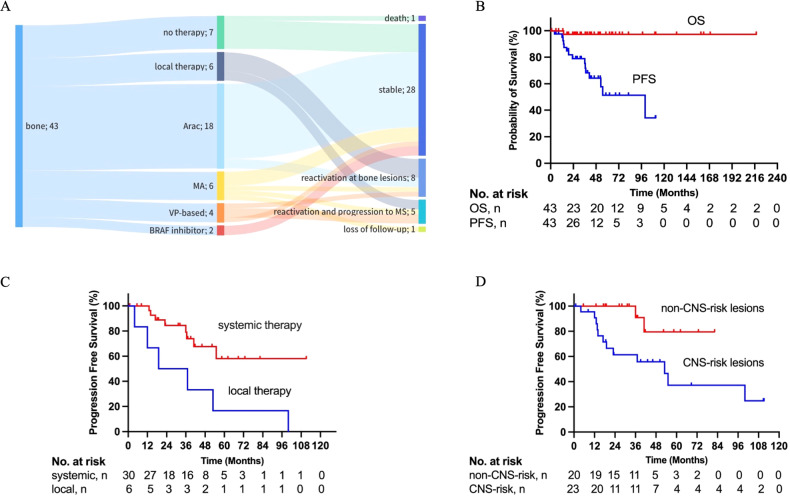
Treatment and outcomes of adult patients with SS-m. **A** The initial treatment and response of LCH in adult with SS-m; **B** Overall survival (OS) and progression-free survival (PFS) of adult patients with SS-m; PFS according to first-line treatment (**C**), and CNS-risk lesions involvement at baseline (**D**).

In our result, we found that all adult patients with SS-m had bone involvement, and the ribs were the most common. Interestingly, we found SS-m LCH had a significantly higher frequency of CNS-risk lesions involvement than SS-s LCH. The discovery of *MAPK* pathway mutations has led to a better understanding of the pathophysiology of LCH. In the present cohort, the proportion of *MAP2K1* mutations and *BRAF* deletions were lower than that in our whole cohort [14] and previous studies [6], whereas the percentage of *BRAF*^*V600E*^ mutations was similar to that of the whole cohort. The overall percentage of *MAPK* pathway mutations in adult LCH with SS-m was lower than that of our previous study [11, 14]. These considerable disparities may have come from crushed bone samples and interference with mutations from decalcification [3]. In addition, in our previous study, we found that *BRAF* deletion was the second most common *MAPK* pathway alternation in adult LCH, and that it strongly correlated with MS LCH in adults [11, 14]. Nevertheless, out of the 11 patients with *BRAF* mutations of SS-m LCH, only one harbored *BRAF* deletion. This supported that *BRAF* deletion is unusual in SS LCH.

The standard first-line treatment for LCH in adults remains undefined, and the treatment depends on symptoms and organ involvement [3, 15]. In this cohort, 69.8% received first-line systemic treatment, and had a better PFS than patients who received local therapy. It also was worth mentioning that both patients who received *BRAF* inhibitors have not reactivated until the last follow-up, but a long-term follow-up was necessary to monitor the future effect and progression. We also proved that non-CNS-risk lesions involvement strongly correlated with better PFS.

In conclusion, we found that bone was the most commonly affected system in adult LCH with SS-m. *BRAF*^*V600E*^ mutation was the most common mutation among LCH in adults with SS-m, while the percentage of *MAP2K1* mutations and *BRAF* deletion was low. First-line systemic treatment and non-CNS-risk lesions involvement predicted better PFS.

### Supplementary information

supplementary material

## Data Availability

The data that support the findings of this study are available from the first author upon reasonable request.

## References

[1] Emile JF, Abla O, Fraitag S, Horne A, Haroche J, Donadieu J (2016). Revised classification of histiocytoses and neoplasms of the macrophage-dendritic cell lineages. Blood.

[2] Allen CE, Merad M, McClain KL (2018). Langerhans-Cell Histiocytosis. N Engl J Med.

[3] Goyal G, Tazi A, Go RS, Rech KL, Picarsic JL, Vassallo R (2022). International expert consensus recommendations for the diagnosis and treatment of Langerhans cell histiocytosis in adults. Blood.

[4] Rigaud C, Barkaoui MA, Thomas C, Bertrand Y, Lambilliotte A, Miron J (2016). Langerhans cell histiocytosis: therapeutic strategy and outcome in a 30-year nationwide cohort of 1478 patients under 18 years of age. Br J Haematol.

[5] Badalian-Very G, Vergilio JA, Degar BA, MacConaill LE, Brandner B, Calicchio ML (2010). Recurrent BRAF mutations in Langerhans cell histiocytosis. Blood.

[6] Nelson DS, van Halteren A, Quispel WT, van den Bos C, Bovée JV, Patel B (2015). MAP2K1 and MAP3K1 mutations in Langerhans cell histiocytosis. Genes Chromosomes Cancer.

[7] Chakraborty R, Burke TM, Hampton OA, Zinn DJ, Lim KP, Abhyankar H (2016). Alternative genetic mechanisms of BRAF activation in Langerhans cell histiocytosis. Blood.

[8] Morimoto A, Ishida Y, Suzuki N, Ohga S, Shioda Y, Okimoto Y (2010). Nationwide survey of single-system single site Langerhans cell histiocytosis in Japan. Pediatr Blood Cancer.

[9] Grois N, Pötschger U, Prosch H, Minkov M, Arico M, Braier J (2006). Risk factors for diabetes insipidus in langerhans cell histiocytosis. Pediatr Blood Cancer.

[10] Khoury JD, Solary E, Abla O, Akkari Y, Alaggio R, Apperley JF (2022). The 5th edition of the World Health Organization Classification of Haematolymphoid Tumours: Myeloid and Histiocytic/Dendritic Neoplasms. Leukemia.

[11] Chen J, Zhao AL, Duan MH, Cai H, Gao XM, Liu T (2022). Diverse kinase alterations and myeloid-associated mutations in adult histiocytosis. Leukemia.

[12] Cao XX, Li J, Zhao AL, He TH, Gao XM, Cai HC (2020). Methotrexate and cytarabine for adult patients with newly diagnosed Langerhans cell histiocytosis: A single arm, single center, prospective phase 2 study. Am J Hematol.

[13] Duan MH, Han X, Li J, Zhang W, Zhu TN, Han B (2016). Comparison of vindesine and prednisone and cyclophosphamide, etoposide, vindesine, and prednisone as first-line treatment for adult Langerhans cell histiocytosis: A single-center retrospective study. Leuk Res.

[14] Cao XX, Duan MH, Zhao AL, Cai H, Chen J, Gao XM (2022). Treatment outcomes and prognostic factors of patients with adult Langerhans cell histiocytosis. Am J Hematol.

[15] Cantu MA, Lupo PJ, Bilgi M, Hicks MJ, Allen CE, McClain KL (2012). Optimal therapy for adults with Langerhans cell histiocytosis bone lesions. PLoS One.

