# Supplementary material for: Clinical characteristics, genomic profiling and outcomes of single system multifocal Langerhans cell histiocytosis in adults with bone involvement
Source: Blood Cancer J. 2023 Sep 5;13(1):135. doi: 10.1038/s41408-023-00913-8 (PMC10480212; doi:10.1038/s41408-023-00913-8)

**Supplementary Tables**

| **Supplementary Table 1. 183 candidate genes for target sequencing** | | | | | |
| --- | --- | --- | --- | --- | --- |
| ALK | ANKRD50 | ANO10 | AP3B1 | **ARAF*** | ARID1B |
| ASXL1 | ATM | ATP4A | ATP9A | ATRX | B2M |
| BCL1 | BCL2L1 | BCOR | BCORL1 | BIRC3 | BOB |
| **BRAF*** | BRD4 | CALR | CASK | CBL | CCDC168 |
| CCR7 | CD274 | CDC73 | CDH1 | CDK6 | CDKN1A |
| CDKN2A | CDKN2B | CEBPA | CEP85 | CHEK2 | CHMP1A |
| COL6A6 | **CSF1R*** | CSF3R | CTNNB1 | DKK2 | DNAH7 |
| DNMT3A | DUSP4 | ECE1 | EFNA4 | **EGFR*** | EMSY |
| EOMES | **ERBB2*** | **ERBB3*** | **ERBB4*** | ETV6 | EZH2 |
| FAS | FBXW7 | FCGBP | FGFR2 | **FGFR3*** | **FLT3*** |
| FOXP3 | GATA1 | GATA2 | GNAQ | GNAS | GREB1L |
| GRK4 | HLA-DQA1 | HLA-DQB2 | HNF1A | HRAS | IDH1 |
| IDH2 | ING1 | ITIH6 | JAK1 | JAK2 | KDM5A |
| KDM6A | **KDR*** | KIF5B | KIR2DL4 | **KIT*** | KMT2B |
| KMT2D | KMT2E | **KRAS*** | LYST | **MAP2K1*** | **MAP3K1*** |
| **MAPK1*** | **MAPK8*** | **MAPK14*** | MDM2 | MDM4 | **MET*** |
| MLH1 | MSH2 | MSH6 | MUNC13-4 | **MYC*** | **MYD88*** |
| NCOA2 | NCOA4 | NCOR2 | NEMO | **NF1*** | NFKB1 |
| NOTCH1 | NOTCH2 | **NRAS*** | NT5DC3 | **NTRK1*** | PAX5 |
| PBRM1 | PBX1 | PCDHA8 | PDCD1 | PDCD1LG2 | PDCD2 |
| **PDGFRA*** | PHIP | PICK1 | PIK3CA | PIK3CD | PIK3R2 |
| PKNOX2 | PLXNA2 | PMS2 | PNISR | POLD1 | POLE |
| PRF1 | PTEN | PTPN11 | RAB27A | **RAF1*** | RB1 |
| RBM10 | RET | RICTOR | ROS1 | RPS6 | RUNX1 |
| RYR2 | SCN1A | SEC62 | SETBP1 | SETD2 | SF3B1 |
| SH2D1A | SHOC2 | SLC10A6 | SLC29A3 | SMAD6 | SMPD1 |
| SOS1 | SPRED1 | SREBF1 | SRSF2 | ST8SIA1 | STAG2 |
| STAT3 | STK11 | STX11 | STXBP2 | TAP2 | TERT |
| TET2 | TLR7 | TLR8 | **TP53*** | TRBV20OR9-2 | TSC1 |
| TTN | U2AF1 | UNC13B | UNC13D | VCL | **VEGFA*** |
| WT1 | XIAP | ZRSR2 |  |  |  |

***The genes associated with MAPK pathway mutations**

**Supplementary Table 2. SS-m LCH patients demographics and clinical characteristics**

| **Characteristics** | **n = 43** |
| --- | --- |
| **Age at diagnosis, years, median (range)** | 34 (21-65） |
| **Sex** |  |
| male, n (%) | 27 (62.8) |
| female, n (%) | 16 (37.2) |
| **Clinical manifestations** |  |
| bone pain, n (%) | 39 (90.7) |
| tumor formation, n (%) | 5 (11.6) |
| toothache, n (%) | 2 (4.6) |
| hearing impairment, n (%) | 1 (2.3) |
| **The median number of bone lesions** | 3 (2-11) |
| **Number of bone lesions** |  |
| 2-5, n (%) | 33 (76.7) |
| 6-10, n (%) | 9 (20.9) |
| >10, n (%) | 1 (2.3) |

**Supplementary Table 3. Invasion site of bone between SS-m LCH and SS-s LCH**

|  | **SS-m LCH (n = 43)** | **SS-s LCH (n = 53)** | ***P* value** |
| --- | --- | --- | --- |
| **Invasion site** |  |  |  |
| ribs n (%) | 22 (51.2) | 27 (50.9) | 0.941 |
| pelvis n (%) | 20 (46.5) | 2 (3.8) | **< 0.0001** |
| spine n (%) | 17 (39.5) | 5 (9.4) | **< 0.0001** |
| skull n (%) | 13 (30.2) | 7 (13.2) | **0.041** |
| maxillofacial bone n (%) | 13 (30.2) | 2 (3.8) | **0.001** |
| limbs n (%) | 12 (27.9) | 7 (13.2) | 0.072 |
| alveolar bone n (%) | 2 (4.6) | 3 (5.7) | 1.000 |
| stemum n (%) | 1 (2.3) | 0 (0.0) | -- |
| CNS-risk lesions n (%) | 23 (53.5) | 12 (22.6) | **0.002** |

SS-m, single system with multifocal; SS-s, single system with unifocal; CNS, central nerve system

**Supplementary Figure legend**

**Supplementary Figure 1.** Percentage of invasion bones of adult LCH patients with SS-m.


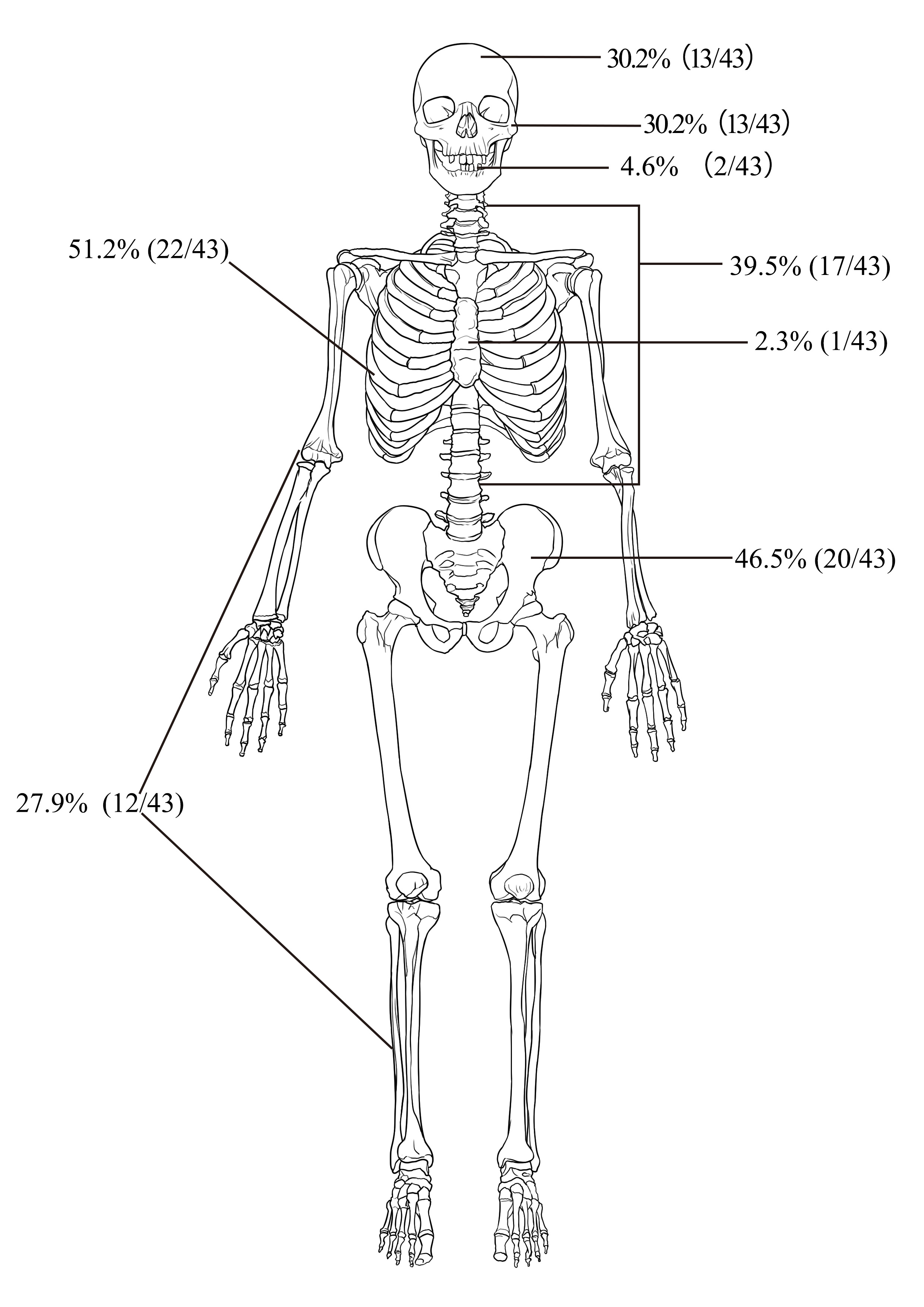

Supplement: Supplementary file 1 — supplementary material [file 41408_2023_913_MOESM1_ESM.docx]
